# Antioxidants prevent particulate matter-induced senescence of lung fibroblasts

**DOI:** 10.1016/j.heliyon.2023.e14179

**Published:** 2023-03-01

**Authors:** Sein Jin, Sung-Jin Yoon, Na-Young Jung, Wang Sik Lee, Jinyoung Jeong, Young-Jun Park, Wantae Kim, Doo-Byoung Oh, Jinho Seo

**Affiliations:** a Aging Convergence Research Center, Korea Research Institute of Bioscience and Biotechnology (KRIBB), Daejeon, 34141, South Korea; b Department of Biochemistry, Chungnam National University, Daejeon, 34134, South Korea; c Environmental Disease Research Center, KRIBB, Daejeon, 34141, South Korea; d Department of Biosystems and Bioengineering, KRIBB School of Biotechnology, University of Science and Technology (UST), Daejeon, 34113, South Korea; e Department of Biomolecular Science, KRIBB School of Bioscience, UST, Daejeon, 34113, South Korea

**Keywords:** Particulate matter, Cellular senescence, Reactive oxygen species, DNA damage Response, Antioxidants

## Abstract

Particulate matter (PM) contributes to human diseases, particularly lung disease; however, the molecular mechanism of its action is yet to be determined. Herein, we found that prolonged PM exposure induced the cellular senescence of normal lung fibroblasts via a DNA damage-mediated response. This PM-induced senescence (PM-IS) was only observed in lung fibroblasts but not in A549 lung adenocarcinoma cells. Mechanistic analysis revealed that reactive oxygen species (ROS) activate the DNA damage response signaling axis, increasing p53 phosphorylation, ultimately leading to cellular senescence via an increase in p21 expression without affecting the p16-pRB pathway. A549 cells, instead, were resistant to PM-IS due to the PM-induced ROS production suppression. Water-soluble antioxidants, such as vitamin C and N-Acetyl Cysteine, were found to alleviate PM-IS by suppressing ROS production, implying that antioxidants are a promising therapeutic intervention for PM-mediated lung pathogenesis.

## 1 Introduction

Industrialization and urbanization have increased environmental pollution, which has harmful effects on human health. Particulate matter (PM), composed of microorganisms, dust, heavy metals, and polycyclic aromatic hydrocarbons (PAHs), is a serious risk factor for various respiratory diseases, such as lung cancer, chronic obstructive pulmonary disease (COPD), and asthma [[1], [2], [3], [4]]. Exposure to PM, has been linked to lung fibrosis in several cohort studies [[5], [6], [7], [8]]. Lung fibrosis is defined as an abnormal accumulation of extracellular matrix caused by persistent mesenchymal activation and impaired epithelial regeneration, which is accompanied by varying degrees of inflammation and eventually results in lung function loss. Among the numerous factors, redox imbalance has been proposed as a key molecular factor in lung fibrosis [[9], [10], [11]]. PM, generated from direct atmospheric release or gas-to-particle translation, is usually classified into PM_10_ (2.5–10 μm), PM_2.5_ (0.1–2.5 μm), and PM_0.1_ (≤0.1 μm), according to its diameter [12]. Fine and ultrafine PM (PM_2.5_ and PM_0.1_) can penetrate into human body barriers, including nasal mucus, lung epithelium, and endothelial walls, and induce severe harmful effects on human health [13]. The World Health Organization (WHO) reported that ambient PM was a significant risk factor for the premature death of 4–9 million people in 2018 [14]. Owing to numerous harmful effects of PM on human health, the WHO classified PM as a first-class carcinogen in 2013, and has regularly issued global air quality guidelines for governments to reduce the risk associated with PM exposure [14,15].

Cellular senescence represents a stable state of cell cycle arrest, which was discovered by Hayflick and Moorhead in 1961 [16,17]. Human fibroblasts cease to divide and enter a proliferatively inactive, but metabolically active state after a fixed number of doubling cycles, referred to as “replicative senescence” or “cellular senescence” in a broad sense. Senescent cells exhibit several hallmarks, including cell cycle arrest, senescence-associated secretory phenotype (SASP), macromolecular damage, and deregulated metabolism [17]. They accumulate in multiple tissues with increasing age and promote aging mainly via SASP, which induces chronic inflammation [18]. Furthermore, senescent cell accumulation in the body is correlated with the increased incidence of aging-related diseases, such as neurodegenerative disorders, diabetes, cardiovascular diseases, osteoarthritis, renal fibrosis, glaucoma, cataract, atherosclerosis, and respiratory diseases [[19], [20], [21]]. According to the inducing factors, cellular senescence can be divided into the following subtypes: replicative senescence (telomere erosion), oncogene-induced senescence (active-oncogene signaling pathways), stress-induced premature senescence (sublethal stress stimuli, such as DNA damage and oxidative stress), therapy-induced senescence (anticancer drug treatment), and post-mitotic cellular senescence (DNA damage in post-mitotic neurons or cardiac myocytes) [22,23]. Recently, a novel pathogenic form of cellular senescence, called virus-induced senescence (VIS), has been added to this subtype list [24]. A broad spectrum of viruses, including the severe acute respiratory syndrome-coronavirus-2 (SARS-CoV-2), lentivirus, adeno-associated virus, and vesicular stomatitis virus, induce cellular senescence upon infection. Lung epithelial cells infected with SARS-CoV-2 secrete SASP factors, including pro-inflammatory cytokines, which attract and activate macrophages, leading to macrophage activation syndrome in the lungs. This suggests that SASP factors generated by VIS critically contribute to the onset of severe coronavirus disease 2019 (COVID-19) lung symptoms [24].

Cellular senescence arrests the cell cycle via two major pathways: p53–p21^Cip1/WAF1^ (p21) and p16^INK4a^ (p16)–pRB pathways [17]. Two key players, p21 and p16, bind to and inhibit the cyclin-dependent kinases (CDKs), CDK2, CDK4, and CDK6, which prevent the transition from G1 to S phase by arresting the cell cycle at G1 phase. DNA damage-induced senescence occurs mainly via the p53–p21 pathway, whereas epigenetically induced senescence is caused by the induction of p16 expression [25]. p53, known as the “guardian of the genome,” initiates the cascade of cellular senescence in response to DNA damage, often caused by telomere attrition, oncogenic stress, and oxidative stress [25].

It was speculated that PM-induced senescence (PM-IS) in lung cells is yet to be reported as most studies used immortalized lung cell lines, including A549 and BEAS-2B [[26], [27], [28], [29], [30], [31], [32], [33], [34]]. Based on this rationale, we observed PM-IS in lung cells for the first time using normal lung fibroblasts instead of immortalized cells. Furthermore, our investigation of the senescence mechanism revealed that antioxidants, such as vitamin C (VitC) and N-acetyl-L-cysteine (NAC), could alleviate PM-IS.

## 2 Materials and methods

### 2.1 Cell culture and supplements

HFL-1 (CCL-153; ATCC, Manassas, VA, USA), IMR-90 (CCL-186; ATCC), and WI-38 (CCL-75; ATCC) human lung fibroblasts and A549 human lung adenocarcinoma cells (CCL-185; ATCC) were used in this study. HFL-1 was cultured in F12–K medium (21127022; Thermo Fisher Scientific, Waltham, MA, USA). IMR90 and WI-38 cells were maintained in Eagle's minimum essential medium (10-009-CV; Corning Cellgro, Manassas, VA, USA). A549 cells were cultured in the Roswell Park Memorial Institute 1640 medium (10-040-CVRC; Corning Cellgro). All media were supplemented with 10% fetal bovine serum (16000044; Thermo Fisher Scientific) and 1% antibiotic-antimycotic (15240062; Thermo Fisher Scientific). Cells were grown in 5% CO_2_ at 37 °C. VitC (l-ascorbic acid; A92902; Sigma-Aldrich, St. Louis, MO, USA) and NAC (A9165; Sigma-Aldrich) were dissolved in distilled water, vitamin D (VitD) (Cholecalciferol; C9756; Sigma-Aldrich) was dissolved in ethanol, and vitamin E (VitE) (DL-α-Tocopherol acetate; T3376; Sigma-Aldrich) was dissolved in chloroform, according to the manufacturer's instructions.

### 2.2 PM_10_ and PM_2.5_ preparation and treatment conditions

European reference material (ERM) CZ-100 (ERMCZ100) was purchased from Sigma-Aldrich. PM_2.5_ was obtained by separating it from the PM_10_ certified reference material (ERM-CZ100) based on the modified sedimentation method [35]. First, 500 mg of ERM-CZ100 was dispersed in 100 mL of ethanol and sonicated (DH.WUC. A03H; Daihan Scientific, Daegu, Korea) for 15 min. The sonicated PM_10_ solution was then sedimented for 30 min at room temperature. After 30 min, 50 mL of the supernatant was collected and centrifuged at 3220×*g* for 5 min (Centrifuge 5810; Eppendorf, Hamburg, Germany) to separate PM_2.5_. After removing the supernatant, PM_2.5_ was collected in a glass vial and dried in a vacuum oven at 80 °C to remove the residual ethanol. Both PM_10_ and PM_2.5_ were dissolved in phosphate-buffered saline (PBS) (LB 001–02; Welgene, Gyeongsan-si, Korea) to make a 10 μg/μL stock solution. One day after plating on the culture dishes, cells were treated with 10 or 25 μg/cm^2^ PM under the same culture conditions (5% CO_2_ at 37 °C) for the indicated days. The culture media of the cells were changed on alternate days. The cells were pre-treated with the indicated concentrations of antioxidants, including NAC, VitC, VitD, and VitE, for 1 h before PM treatment. Furthermore, they were supplemented into the media whenever the media were changed.

### 2.3 Cell proliferation analysis

Cell proliferation was measured using the CellTiter-Glo 2.0 assay kit (G9243; Promega, Madison, WI, USA) and cell counting method. Cells were plated on a 96-well black plate for the CellTiter-Glo 2.0 assay, whereas a 24-well plate was used for cell counting. After seeding the cells for 24 h, they were treated with the indicated concentrations of PM, followed by either CellTiter-Glo 2.0 treatment or direct counting using a hemocytometer. The luminescence intensity of cells was measured using SpectraMax i3 (Molecular Devices, San Jose, CA, USA).

### 2.4 Senescence-associated β-galactosidase (SA-β-gal) staining

SA-β-Gal staining was performed using a commercial senescence detection kit (K320; Biovision, Milpitas, CA, USA). Briefly, cells were seeded in a six-well plate and subjected to PM treatment for 24 h after cell seeding. The culture medium was changed every 2 days for 7 days. After fixation, the cells were stained with a solution mix containing X-gal. Images of SA-β-Gal-stained cells were obtained using an inverted microscope (CKX53; Olympus, Tokyo, Japan).

### 2.5 Immunoblotting and antibodies

Cells were lysed with the sample buffer and boiled for 10 min. Samples were separated by sodium dodecyl sulfate–polyacrylamide gel electrophoresis and transferred onto a polyvinylidene fluoride membrane (IPVH00010; Merck Millipore, Burlington, MA, USA). The membrane was incubated with 5% skim milk for 30 min and then incubated with primary antibodies at 4 °C overnight. After incubation with the primary antibodies, the membrane was washed with Tris-buffered saline containing 0.1% Tween-20, and then incubated with the horseradish peroxidase (HRP)-conjugated goat anti-rabbit IgG secondary antibody (ADI-SAB-300-J; 1:3,000; Enzo Life Sciences, Farmingdale, NY, USA) or HRP-conjugated goat anti-mouse IgG secondary antibody (G-21040; 1:3,000; Thermo Fisher Scientific) for 1 h. Protein bands were visualized using a chemiluminescence imaging system (FUSION Solo X; VILBER, Collégien, France). The following primary antibodies were used: p53 (sc-126; 1:1,000; Santa Cruz Biotechnology, Dallas, TX, USA), phospho-p53 (9284S; 1:1,000; Cell Signaling Technology), p21 (2947S; 1:1,000; Cell Signaling Technology, Danvers, MA, USA), Rb (sc-102; 1:1,000; Santa Cruz Biotechnology), phospho-Rb (9308S; 1:1,000; Cell Signaling Technology), p16 (A301-267A; 1:1,000; Thermo Fisher Scientific), Ataxia telangiectasia mutated (ATM) (ab32420; 1:1,000; Abcam, Cambridge, UK), phospho-ATM (ab81292; 1:1,000; Abcam), and β-actin (A5316; 1:5,000; Sigma-Aldrich). Image J (Version 1.52a; NIH, Bethesda, MD, USA) was utilized to compute the intensities of protein bands, followed by relative amounts normalized to β-actin (target protein/β-actin) [36].

### 2.6 Reverse transcription-quantitative real-time polymerase chain reaction (RT-qPCR)

Total RNA was isolated from the cells using the Hybrid-R kit (305-101; GeneAll, Seoul, Korea), according to the manufacturer's instructions. cDNA synthesis was performed using M-MLV reverse transcriptase (28025-013; Thermo Fisher Scientific), random hexamers (N8080127; Thermo Fisher Scientific), and dNTPs (R0192; Thermo Fisher Scientific). After cDNA synthesis, qPCR was performed to determine the levels of target mRNAs using a qPCR kit (204143; QIAGEN, Hilden, Germany) and Rotor-Gene Q machine (R0310221; QIAGEN), according to the manufacturer's instructions. Relative mRNA expression was assessed using the following formula: Relative mRNA = 2^(ΔCT(target)−ΔCT(Gapdh))^.

The sequences of qPCR primers for each gene are listed below: glyceraldehyde‐3‐phosphate dehydrogenase (*GAPDH*): Fw 5′-GATCATCAGCAATGCCTCCT-3′ and Rv 5′-TGTGGTCATGAGTCCTTCCA-3′; *p21*: Fw 5′-GACTCTCAGGGTCGAAAACG-3′ and Rv 5′-GGCGTTTGGAGTGGTAGAAA-3′; *p53*: Fw 5′-AACCCACAGCTGCACAG-3′ and Rv 5′-CCTTCCCAGAAAACCTACCAG-3′; *p14*^*ARF*^ Fw 5′-CTCAGTAGCATCAGCACGAG-3′ and Rv 5′-TGGTGACCCTCCGGATT-3′; *p16*^*INK4a*^ Fw 5′-ACCCTGTCCCTCAAATCCT-3′ and Rv 5′-GTGCCACATTCGCTAAGTG-3′; *Rb* Fw 5′-TGTGAACATCGAATCATGGAA-3′ and Rv 5′-TGATCAGTTGGTCCTTCTTCTCG-3′; interleukin (*IL*)-6 Fw 5′- GCACTGGCAGAAAACAACCT-3′ and Rv 5′-TCAAACTCCAAAAGACCAGTGA-3′; *IL-8* Fw 5′-CTCTTGGCAGCCTTCCTGATT-3′ and Rv 5′-ACTCTCAATCACTCTCAGTTCT-3′; C–C motif chemokine ligand 2 (*CCL2*) Fw 5′-CAGCAGCAAGTGTCCCAAAG-3′ and Rv 5′-TTGGCCACAATGGTCTTGAA-3′; matrix metalloproteinase (*MMP*)-*3* Fw 5′-CTCCAACCGTGAGGAAAATC-3′ and Rv 5′-CATGGAATTTCTCTTCTCATCAAA-3′; tumor necrosis factor (*TNF*)-*α* Fw 5′-GCCCAGGCAGTCAGATCATCT-3′ and Rv 5′-TTGAGGGTTTGCTACAACATGG-3′.

### 2.7 Confocal microscope analysis

γH2AX confocal microscopy experiments were conducted as previously reported with a slight modification [37,38]. The cells were plated on 50 mm μ-dish (81136; ibidi, Munchen, Germany). After 24 h of cell seeding, the cells were treated with PM, as described above. The cells were fixed with 4% paraformaldehyde (IBS-BP031; iNtRON Biotechnology, Seongnam-Si, Korea) and permeabilized via incubation with PBS containing 0.05% Triton-X 100. Permeabilized cells were blocked via incubation with 2.5% bovine serum albumin for 30 min, followed by incubation with the primary γH2AX antibody (ab81299; 1:100; Abcam), at 4 °C overnight. The resulting samples were incubated with goat anti-rabbit IgG Alexa Fluor 488 antibody (A-11034; 1:200; Thermo Fisher Scientific) for 1 h. Stained cells were observed under a confocal microscope (LSM 880; Carl Zeiss, Oberkochen, Germany).

### 2.8 Reactive oxygen species (ROS) analysis

Cellular ROS levels were determined using chloromethyl derivative of H_2_DCFDA (CM-H_2_DCFDA; C6827; Thermo Fisher Scientific), according to the manufacturer's instructions. Briefly, control cells or PM-treated cells were washed thrice with Hanks' balanced salt solution (14025092; Thermo Fisher Scientific) and resuspended in 10 μM CM-H_2_DCFDA. The cells were incubated for 45 min in the dark. Propidium iodide (P4170; Sigma-Aldrich) was added to distinguish the live cells from dead cells. Stained cells were analyzed using flow cytometers (BD FACS Verse; BD Bioscience, Franklin Lakes, NJ, USA).

### 2.9 Statistical analysis

All statistical analyses were computed using GraphPad Prism software (Ver. 9.4.1; La Jolla, CA, USA). When only two groups were analyzed (RT-qPCR), a Student's t-test was utilized to determine statistical differences and significance. One-way ANOVA with Tukey's HSD was used in CM-H2DCFDA analyses, whereas two-way ANOVA with Tukey's HSD was used in cell viability, cell counting, and vitamin C treatment analysis. Error bars represented standard deviation (SD).

## 3 Results

### 3.1 PM treatment induces the cellular senescence of normal lung fibroblasts

**Fig. 1:**
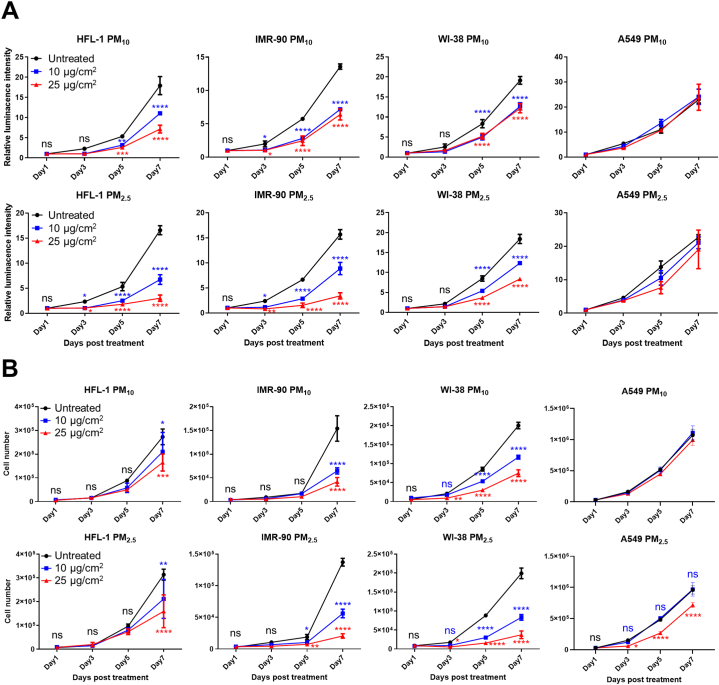
Sublethal and prolonged exposure to particulate matter (PM)_10_ and PM_2.5_ inhibits the proliferation of lung fibroblasts. IMR-90, HFL-1, and WI-38 cells were treated with 10 or 25 μg/cm^2^ of PM_10_ or PM_2.5_, while control groups were untreated. Cell proliferation was measured on days 1, 3, 5, and 7 after PM treatment. Cell proliferation was analyzed using CellTiter-Glo (A) and hemocytometer (B). Data are presented as the mean ± standard deviation (SD) of three independent experiments. Statistical analyses were calculated by two-way ANOVA. Significances were determined by Tukey's HSD (ns = not significant; *P < 0.05, **P < 0.01, ***P < 0.001, and ****P < 0.0001).

A comparative study was conducted using three lung fibroblast cell lines (HFL-1, IMR-90, and WI-38) and a lung adenocarcinoma cell line (A549) to determine whether PM treatment exerted different effects on the proliferation of these cells. Although both immortalized A549 and BEAS-2B cells have been widely used in previous studies, the BEAS-2B cell line was excluded as it expresses the SV40 T-antigen, disrupting the cellular signaling pathway by forming a complex with p53 and pRB [[39], [40], [41]]. Various conditions of PM treatments were tested to observe their prolonged exposure effects under sublethal doses, and the following treatment conditions were selected: 10 or 25 μg/cm^2^ PM treatment for 1 week (Fig. S1). PM_10_ and PM_2.5_ treatments significantly inhibited the proliferation of all lung fibroblasts in the luminescent cell viability (CellTiter-Glo) assay, but they did not affect the proliferation of A549 cells (Fig. 1A). Notably, PM_2.5_ treatment showed a much more significant inhibition (∼80%) of cell proliferation than PM_10_ (∼50% inhibition) (Fig. 1A). To crosscheck the results of the CellTiter-Glo assay, the cell numbers were counted directly using a hemocytometer, which excluded the possibility of cell viability reduction caused by cell death. Direct cell counting results clearly showed that PM_10_ and PM_2.5_ treatments reduced the number of proliferated cells in all lung fibroblasts (Fig. 1B). Although 25 μg/cm^2^ PM_2.5_ treatment slightly reduced the proliferation of A549 cells on days 5 and 7 in a direct cell counting experiment, the other treatment conditions (10 or 25 μg/cm^2^ PM_10_ and 10 μg/cm^2^ PM_2.5_) did not inhibit A549 cell proliferation (Fig. 1B).

**Fig. 2:**
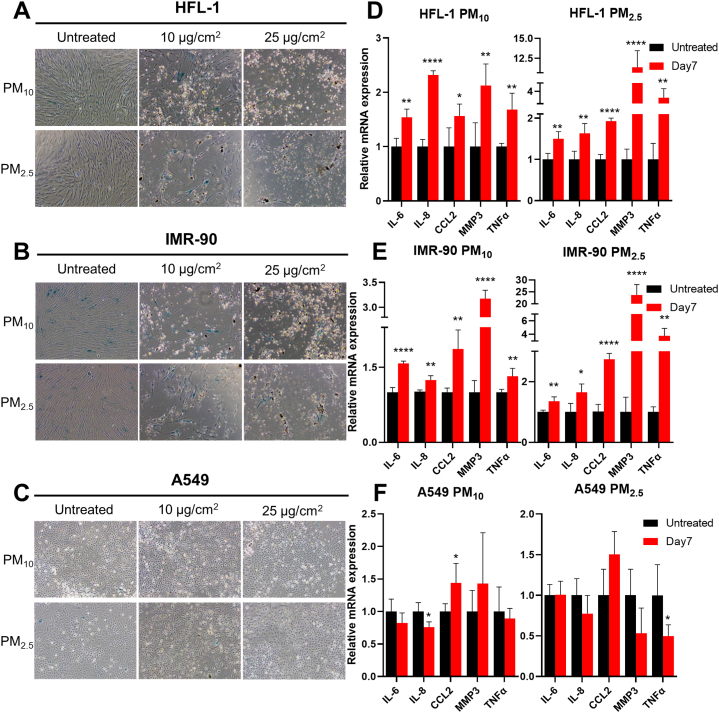
PM treatment promotes the cellular senescence of lung fibroblasts. (A–C) Cells were treated with the indicated concentration of PM_10_ or PM_2.5_, while control groups were untreated. After 7 days of PM treatment, SA-β-gal staining was performed, and the cells were observed under a microscope. (D–E) In RT-qPCR experiments for SASP gene expression analysis, 10 μg/cm^2^ of PM_10_ or PM_2.5_ was treated for 7 days, while control groups were untreated for the same days. Relative fold changes of mRNA expression are presented as the mean ± SD of four independent experiments (ns = not significant; *p < 0.05, **p < 0.01, and ***p < 0.001).

We suspected that the proliferatively inactive state of lung fibroblasts corresponded to cellular senescence as it did not accompany cell death, implying a metabolically active state [23]. Moreover, PM-treated lung fibroblasts displayed flattened and enlarged morphologies that are characteristic of senescent cells. SA-β-gal staining assay was performed to confirm the senescent state of lung fibroblasts. Treatment with PM_10_ and PM_2.5_ increased the population of SA-β-gal-positive cells in all lung fibroblasts, whereas SA-β-gal-positive cells were rarely observed in A549 cells (Fig. 2A–C). Notably, although A549 proliferation was slightly retarded in the 25 μg/cm^2^ PM_2.5_ treatment group (Fig. 1B), SA-β-gal-positive cells did not increase under the same conditions (Fig. 2C).

Next, we checked the changes in the expression levels of SASP factors using RT-qPCR. PM_10_ and PM_2.5_ treatments increased the mRNA expression levels of many SASP factors, including IL-6, IL-8, CCL2, MMP3, and TNF-α, in lung fibroblasts but not in A549 cells (Fig. 2D–F). These results indicate that PM treatment induces the cellular senescence of lung fibroblasts with inhibition of cell proliferation, but the same conditions do not induce the cellular senescence of A549 cells.

### 3.2 PM-IS of lung fibroblasts occurs via the p53–p21 signaling pathway

**Fig. 3:**
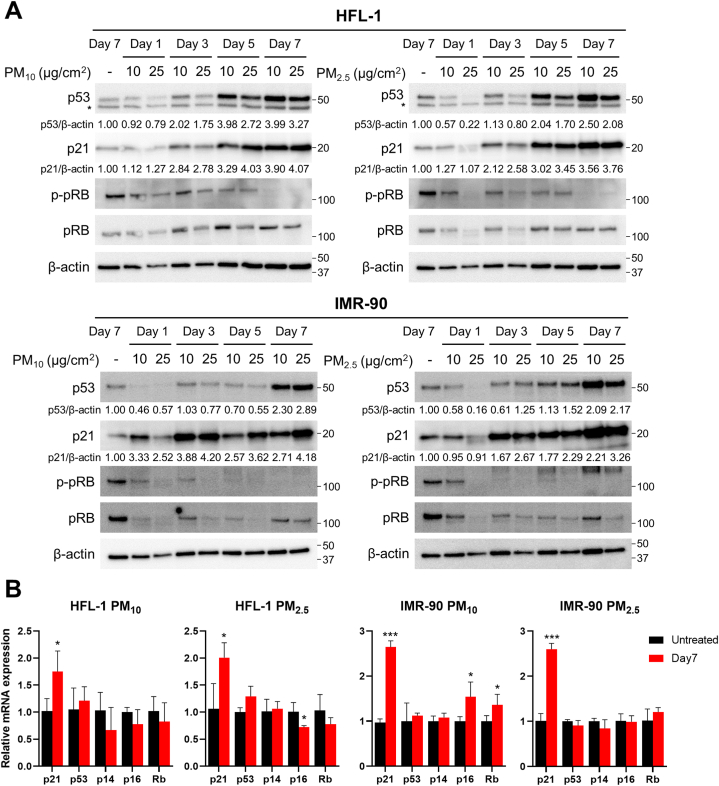
PM induces p21 mRNA expression by activating the p53–p21 signaling axis. (A) Cells were treated with 10 or 25 μg/cm^2^ of PM_10_ or PM_2.5_ for the indicated days, while control groups were untreated for 7 days. Harvested cells were analyzed using anti-p53, -p21, -p-pRB, and -pRB antibodies. The asterisks denote non-specific bands, which were suspected to be β-actin bands remaining after the extensive stripping and washing. (B) HFL-1 and IMR-90 cells were treated with 10 μg/cm^2^ of PM_10_ or PM_2.5_ for 7 days, while control groups were untreated for the same days. mRNA expression levels of senescence driver genes were determined via RT-qPCR analysis. Relative fold changes of mRNA expression are presented as the mean ± SD of three independent experiments (ns = not significant; *p < 0.05, **p < 0.01, and ***p < 0.001). Images of Western blot raw data are presented in Fig. S3.

We investigated the changes in the expression of key senescence drivers during PM-IS in lung fibroblasts. The best-characterized senescence drivers are p53, p21, p16, and pRB, which permanently arrest the cell cycle by directly or indirectly regulating CDK functions [17]. Immunoblot analysis showed that the protein levels of p53 and p21 increased in a time-dependent manner over 7-day PM treatment (Fig. 3A). In contrast, pRB and phosphorylated pRB (p-pRB) amounts did not change (Fig. 3A), and p16 was not detected under our experimental conditions, possibly due to a trivial level of expression (data not shown). We also observed that the p53 and p21 expression levels of PM treatment groups on day 1 were lower than those of untreated groups on day 7. Therefore, it was speculated that the prolonged culture without PM treatment would slightly increase p53 and p21 expressions.

RT-qPCR experiments were performed to check whether the expression of key senescence driver proteins was regulated at the transcriptional level (Fig. 3B). Significant increase (1.8–2.7 fold) in p21 mRNA levels was observed in PM_10_-and PM_2.5_-treated lung fibroblasts, indicating a correlation between PM-IS and transcriptional upregulation of p21. In contrast with the p53 protein increase observed by immunoblotting (Fig. 3A), p53 mRNA levels rarely changed during PM-IS (Fig. 3B), implying the existence of post-transcriptional regulation, such as the prevention of p53 protein degradation. The other mRNA levels (p14, p16, and RB) showed mostly insignificant changes (Fig. 3B). Although statistically significant increases in p16 and RB mRNA levels were observed only in PM_10_-treated IMR-90 cells, the values of the cycle thresholds were 31–34, which suggested possible noise resulting from meager amounts. In summary, PM-IS of lung fibroblasts is mainly mediated by the p53–p21 signaling pathway, which is accompanied by the transcriptional upregulation of p21.

### 3.3 PM-induced oxidative stress results in DNA damage, followed by ATM–p53–p21 signaling activation

**Fig. 4:**
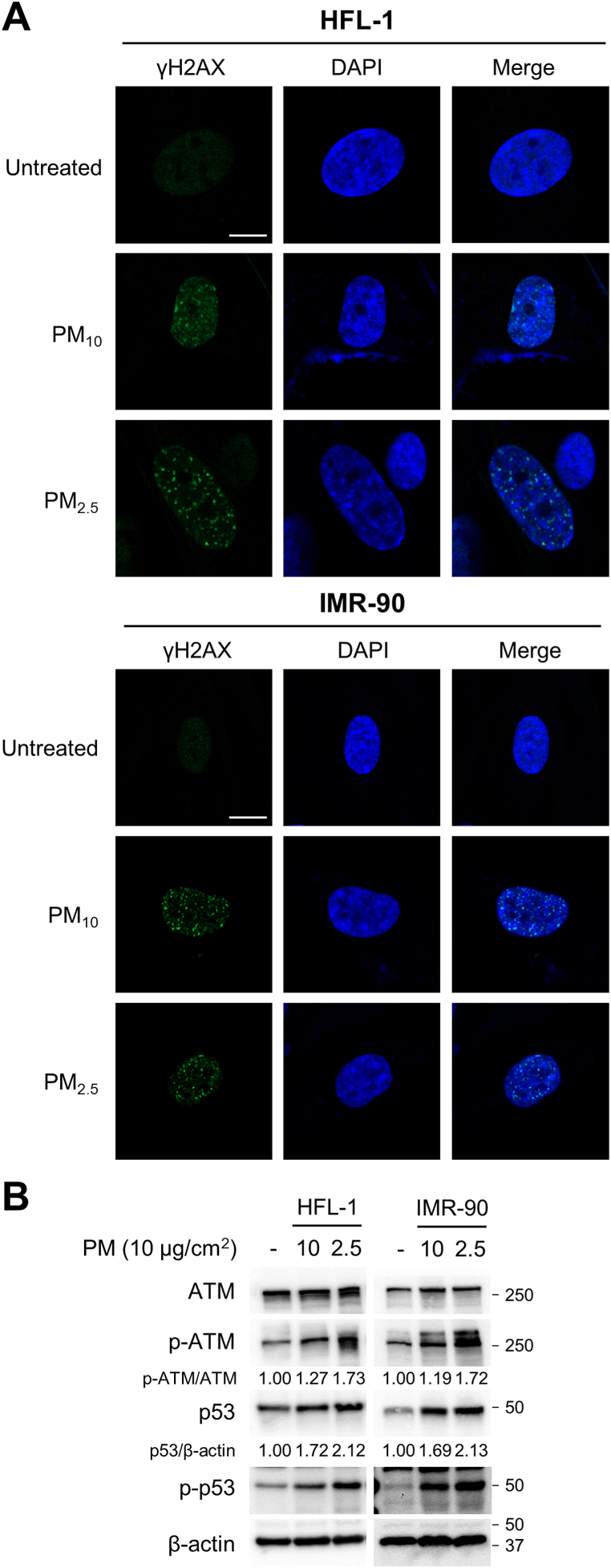
Prolonged exposure to PM causes DNA damage in lung fibroblasts. Cells were treated with 10 μg/cm^2^ of PM_10_ or PM_2.5_ for 7 days, while control groups were untreated for the same days. (A) γH2AX and the nucleus were stained with the anti-γH2AX antibody and 4′,6-diamidino-2-phenylindole dihydrochloride (DAPI), respectively. Stained cells were observed under a confocal microscope. The scale bars are 10 μm. (B) DNA damage response (DDR) signaling pathway was analyzed using anti-p-ATM, -ATM, -p-p53, and -p53 antibodies. Images of Western blot raw data are presented in Fig. S4.

Phosphorylated-H2AX (γH2AX) staining assay was performed to analyze DNA damage in PM-treated lung fibroblasts because the p53–p21 signaling pathway is mainly activated in response to DNA damage [42]. Lung fibroblasts treated with 10 μg/cm^2^ PM_10_ and PM_2.5_ for 7 days showed an increased population of γH2AX-positive cells (Fig. 4A). In particular, PM_2.5_ treatment more greatly increased the γH2AX foci numbers in the fibroblast nucleus as well as the γH2AX-positive cell numbers (Fig. 4A), indicating that PM_2.5_ treatment caused more severe DNA damage than PM_10_.

Next, we investigated whether PM-induced DNA damage activates the DNA damage response (DDR) signaling pathway. ATM, one of the most upstream kinases in DDR signaling, is phosphorylated in response to DNA double-strand breaks (DSBs). Fig. 4B shows that phosphorylated ATMs were upregulated in PM_10_-and PM_2.5_-treated lung fibroblasts. Since phosphorylated ATM phosphorylates downstream signaling molecules, including p53, Chk1, and Chk2, we further examined p53 phosphorylation. As expected, p53 phosphorylation was upregulated in PM_10_-and PM_2.5_-treated lung fibroblasts (Fig. 4B). This result implied that PM-induced DNA damage activated ATM**–**p53**–**p21 signaling, which turned on the DDR signaling pathway.

**Fig. 5:**
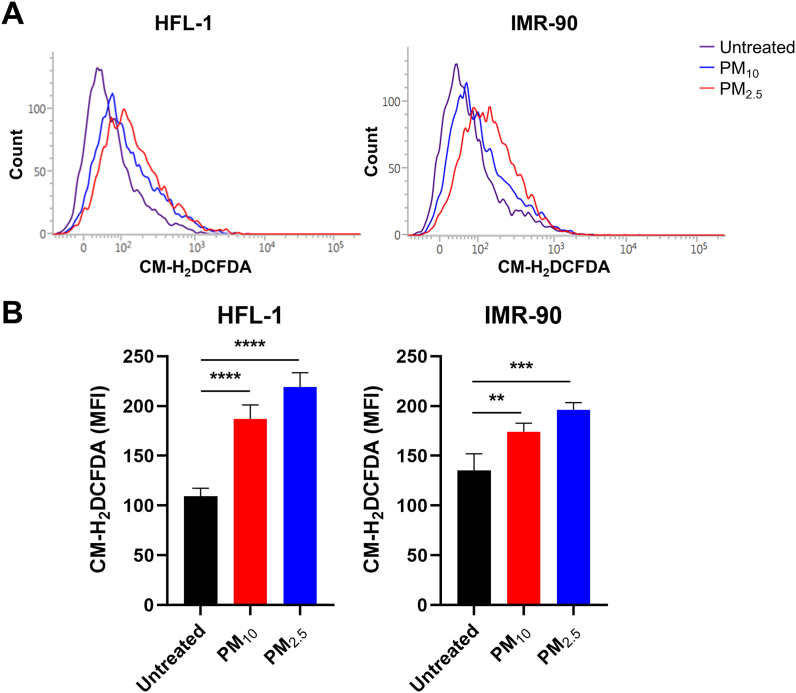
PM treatment produces excessive reactive oxygen species (ROS) in lung fibroblasts. Cells were treated with 10 μg/cm^2^ of PM_10_ or PM_2.5_ for 7 days, while control groups were untreated for the same days. (A) ROS were detected via chloromethyl derivative of H_2_DCFDA (CM-H_2_DCFDA) staining-based (10 μM) flow cytometry analysis. (B) Data are presented as the mean ± SD of RFI of three independent experiments. Statistical analyses were performed by one-way ANOVA. Significances were calculated by Tukey's HSD (**P < 0.01, ***P < 0.001, and ****P < 0.0001).

We investigated how PM induced DNA damage in lung fibroblasts. PMs comprising PAHs and persistent organic pollutants on their surfaces have been reported to activate the aryl hydrocarbon receptor (AHR), which generates excessive ROS via CYP1A1 induction [43,44]. In addition, PMs contain numerous redox-active metals (e.g., Fe, Cu, Ni, Zn, Cr, As, and Mn) that produce ROS through Fenton's reaction [45]. Therefore, we analyzed cellular ROS levels using a CM-H_2_DCFDA assay to determine whether PM treatment conditions produced excessive ROS. Fig. 5A and B shows that PM_10_ and PM_2.5_ treatments significantly increased ROS levels (1.3–2 fold) in lung fibroblasts. Notably, the PM_2.5_ treatment produced more ROS than PM_10_ (Fig. 5A and B). This is in good agreement with our observation that PM_2.5_ induced stronger DDR and more severe senescence phenotypes in lung fibroblasts.

Collectively, PM treatment produced excessive ROS in lung fibroblasts, which caused DNA damage, followed by activation of the ATM**–**p53**–**p21 signaling pathway for defense against DSBs.

### 3.4 A549 lung adenocarcinoma cells have the unique ability to counteract PM-induced oxidative stress

**Fig. 6:**
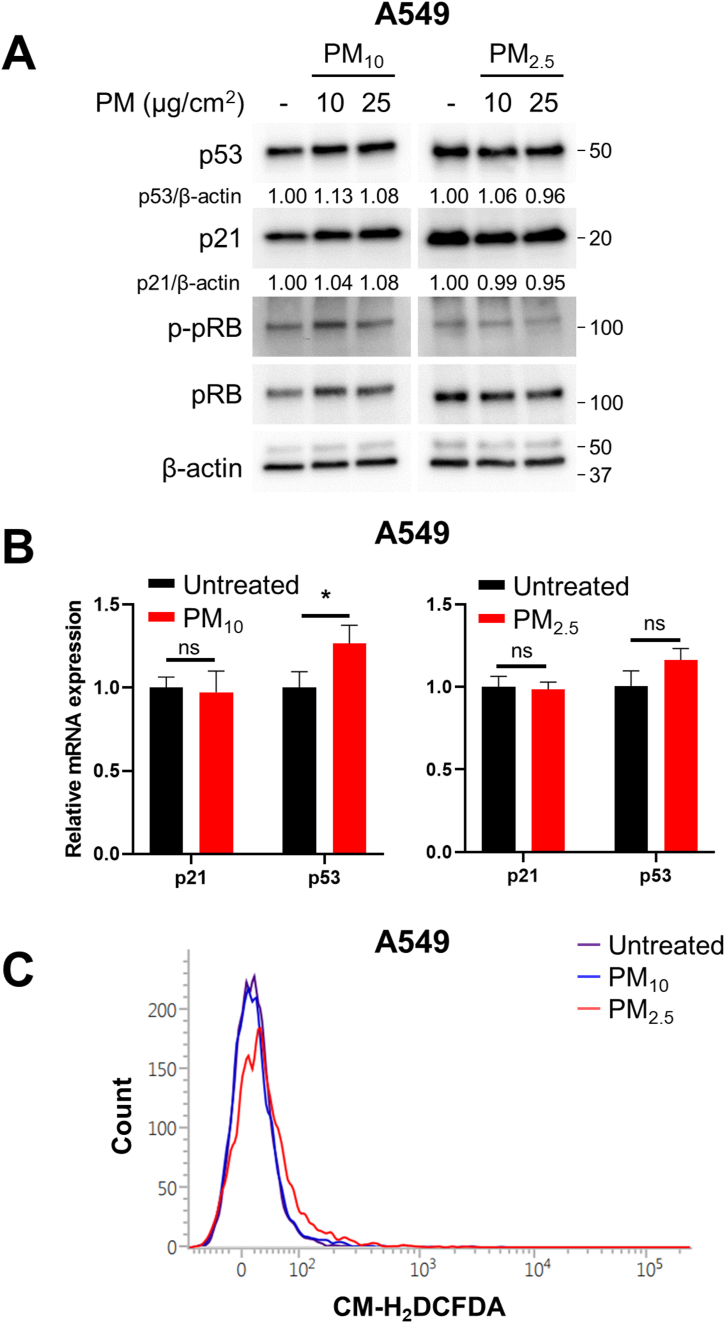
A549 cells are resistant to PM-induced cellular senescence (PM-IS) by suppressing excessive ROS production. A549 cells were treated with 10 or 25 μg/cm^2^ of PM_10_ or PM_2.5_ for 7 days, while control groups were untreated for the same days. (A) Expression levels of senescence driver proteins were determined via immunoblotting. (B) p21 and p53 mRNA expression levels were determined via RT-qPCR analysis after treatment with 10 μg/cm^2^ of PM. Relative fold changes are presented as the mean ± SD of three independent experiments. Statistical analyses were performed by Student-t-test (ns = not significant; *P < 0.05). (C) ROS production was analyzed via CM-H_2_DCFDA staining-based (10 μM) flow cytometry after treatment with 10 μg/cm^2^ of PM. Images of Western blot raw data are presented in Fig. S5.

A549 lung adenocarcinoma cells have been widely used in many studies to investigate harmful effects of PM. However, the results obtained using A549 cells differed markedly from those obtained using lung fibroblasts. The PM treatment condition inducing senescence of lung fibroblasts did not cause significant changes in A549 cell proliferation (Fig. 1), SA-β-gal staining, or SASPs expression (Fig. 2). These results raise the question of what makes A549 cells resistant to PM-IS. First, we analyzed changes in senescence driver proteins in A549 cells. The PM_10_ and PM_2.5_ treatments did not change the protein (Fig. 6A) or mRNA levels (Fig. 6B) of senescence drivers (p53, p21, p-pRb, and pRb). These results suggest that A549 cells have a unique (different from lung fibroblasts) mechanism upstream of the cellular senescence signaling pathway. Therefore, we speculate that this unique mechanism could be explained by the KRAS G12S mutation, which results in a constitutively active KRAS signaling pathway that can counteract ROS production by regulating mitochondrial ROS production enzymes and diverse oxidative stress defense mechanisms [[46], [47], [48], [49], [50], [51], [52], [53], [54], [55], [56], [57]]. Based on this speculation, we hypothesized that ROS production would be suppressed in A549 cells owing to active KRAS signaling. As expected, the PM_10_ and PM_2.5_ treatments barely changed the DCF intensity of A549 cells (Fig. 6C), which is in sharp contrast to PM-treated lung fibroblasts (Fig. 5). In summary, A549 cells were resistant to PM-IS because of their unique ability to suppress ROS production.

### 3.5 Antioxidants alleviate PM-IS of lung fibroblasts by reducing excessive ROS production

**Fig. 7:**
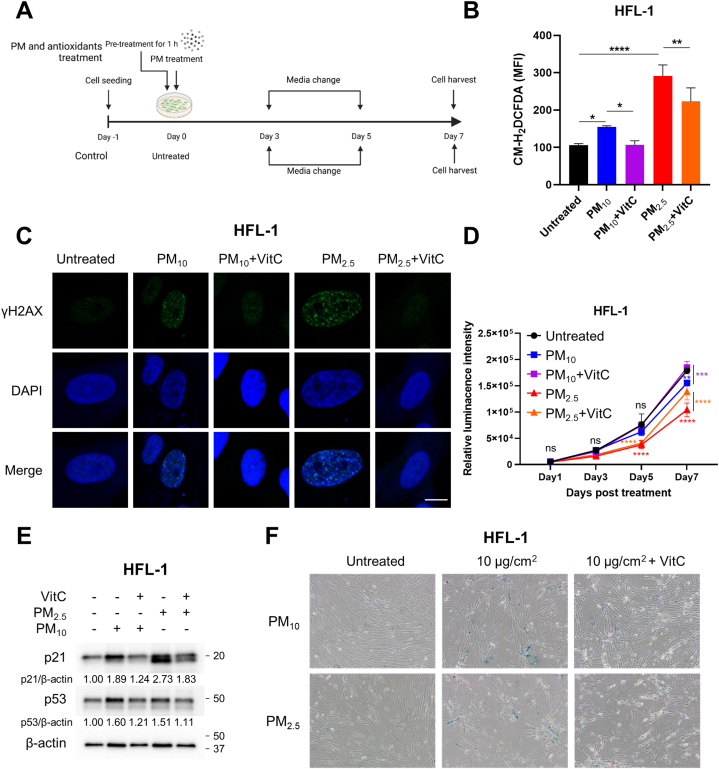
Vitamin C (VitC) prevents PM-IS of lung fibroblasts by removing PM-induced ROS. (A) Schematic diagram of the antioxidant treatment experiment. Cells were pre-incubated with 100 μM VitC for 1 h, followed by treatment with 10 μg/cm^2^ of PM_10_ or PM_2.5_ for the indicated days, while control groups were untreated for 7 days. (B) ROS production was analyzed via CM-H_2_DCFDA staining-based (10 μM) flow cytometry. Data are presented as the mean ± SD of RFI from four independent experiments. Statistical analysis was performed by one-way ANOVA. Significances were calculated by Tukey's HSD (*P < 0.05, **P < 0.01, and ****P < 0.0001). (C) γH2AX and nucleus were stained with the anti-γH2AX antibody and DAPI, respectively, followed by confocal microscope analysis. The scale bar is 10 μm. (D) Cell proliferation was measured using CellTiter-Glo on days 1, 3, 5, and 7 after PM treatment. Data are presented as the mean ± SD of three independent experiments. Statistical analysis was performed by two-way ANOVA. Significances were calculated by Tukey's HSD (ns = not significant; **P < 0.01, ***P < 0.001, and ****P < 0.0001). (E) Senescence driver protein levels were determined via immunoblotting using anti-p53 and -p21 antibodies. (F) SA-β-gal staining was performed, and the cells were observed under a microscope. Images of Western blot raw data are presented in Fig. S6.

Because A549 cells with ROS suppression ability were resistant to PM-IS, we attempted to identify an antioxidant to alleviate PM-induced oxidative stress. Available antioxidants (NAC, VitC, VitD, and VitE) were screened to identify the best one to alleviate PM-IS in lung fibroblasts (Fig. S2). Antioxidants were pre-treated before PM treatment, and the media exchanged on alternate days were also supplemented with the same antioxidant (Fig. 7A). As shown in Fig. S2, VitC and NAC effectively reduced the excess ROS produced by PM treatment, whereas VitD and VitE did not. Since VitC can be taken daily without worrying about side effects, we focused on VitC for further experiments. Since VitC significantly reduced PM-induced ROS production in lung fibroblasts (Fig. 7B), it dramatically decreased γH2AX-positive populations compared to PM-treated fibroblasts (Fig. 7C). PM-induced growth retardation of lung fibroblasts was rescued by VitC treatment (Fig. 7D). Furthermore, VitC blocked PM-induced elevation of p21 expression (Fig. 7E), suggesting that it suppresses DNA damage by scavenging excessive ROS. Finally, we investigated whether VitC could prevent PM-IS in lung fibroblasts. Fig. 7F shows that VitC treatment decreased the SA-β-gal-positive population in PM_10_-and PM_2.5_-treated lung fibroblasts.

**Fig. 8:**
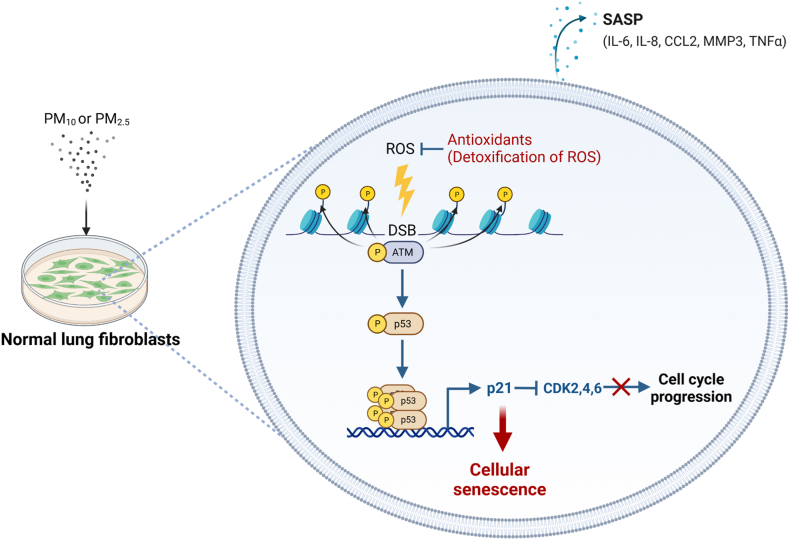
Schematic diagram of PM-IS mechanism. Exposure to PM_10_ and PM_2.5_ causes DNA double-strand break (DSB) by producing excessive ROS. DSB activates the DDR signaling pathway, ATM–p53–p21, which induces cellular senescence by inhibiting the cyclin-dependent kinases (CDKs). VitC prevents PM-IS by removing excess ROS.

In conclusion, antioxidants, such as VitC, prevented PM-IS in lung fibroblasts by removing the excess ROS (Fig. 8).

## 4 Discussion

Many cohort studies have established a significant correlation between PM exposure and respiratory diseases. However, the molecular mechanism by which PM exposure induces respiratory diseases has not yet been elucidated. With the eventual aim to reveal this mechanism, we planned experiments and found that only a few studies have used an appropriate cellular model. Most studies have used immortalized cell lines, such as A549 (lung adenocarcinoma) and BEAS-2B (lung epithelial cells), under acute PM treatment conditions [[26], [27], [28], [29], [30], [31], [32], [33], [34]]. Immortalized cell lines have the capability of unlimited proliferation, which is different from normal cells with Hayflick's limit of proliferation. Thus, we postulated that normal lung fibroblasts could be employed as a better cellular model that mimics real lung conditions. Therefore, comparative experiments were performed using normal lung fibroblasts (HFL-1, IMR-90, and WI-38) and an immortalized A549 cell line. In addition, the experimental conditions of prolonged and sublethal PM treatments were carefully designed and established to mimic real PM exposure in everyday life. The established PM treatment conditions induced cellular senescence in normal lung fibroblasts, but they hardly affected the A549 cell proliferation. This is the first study to observe the PM-IS of lung cells, although PM-IS of skin fibroblasts has been previously reported [58].

The discovery of PM-IS in lung fibroblasts prompted us to investigate its molecular mechanisms as a next step. Our study clarified that PM-IS in lung fibroblasts mainly occurs through the p53-p21 signaling pathway (Fig. 8), which contrasts with the p16-dependent PM-IS in skin fibroblasts [58]. In lung fibroblasts, PM-induced ROS production causes DNA damage, such as DSBs, which activate ATM phosphorylation. In succession, phosphorylated ATM activates p53 downstream signaling, including the transcriptional upregulation of p21. This ATM**–**p53**–**p21 signaling cascade inhibits CDKs and prevents cell cycle progression, which finally results in cellular senescence. We also showed that A549 cells did not activate ATM**–**p53**–**p21 signaling for cellular senescence under the same PM treatment conditions because they could suppress PM-induced ROS production.

PM-IS can be regarded as a cellular defense mechanism from the point of view that it is induced to counteract DNA damage stress caused by PM-induced ROS. However, cellular senescence often generates SASP factors that can cause detrimental inflammation, dysregulated immune reactions, and pathogenic symptoms. For example, SASP elevation by skin damage–induced senescence in older adults attracts CCR2^+^CD14^+^ monocytes that secrete prostaglandin E, which inhibits T cell response and antigen-specific immunity [59]. In the case of VIS caused by SARS-CoV-2 infection, senescent cells secrete multiple SASP factors, attracting macrophages into the lungs and polarizing them into the pro-inflammatory M1 type [24]. Subsequently, these infiltrated pro-inflammatory macrophages further induced SASP-amplifying secondary senescence of endothelial cells, which led to severe COVID-19 symptom appearances, such as endothelial damage and widespread thrombosis in the infected lung tissue [24]. In this study, we also observed that PM-IS in lung fibroblasts increased the expression of SASP factors, including IL-6, IL-8, CCL2, MMP3, and TNFα. Increased IL-6 expression is consistent with a previous report that it is a promising blood biomarker associated with PM exposure [60]. Although PM exposure is related to inflammatory cytokines [61], it remains unclear how it induces inflammatory cytokines. Our observation of PM-IS-mediated SASP provides a clue for solving this missing link: PM-induced senescent lung cells secrete SASP factors, including inflammatory cytokines. These SASP factors attract monocytes and macrophages to infiltrate the lungs, which is followed by pro-inflammatory reprogramming. Chronic inflammation in the lung contributes to the development of respiratory diseases, such as COPD, asthma, and lung cancer. Currently, we are preparing a mouse model to confirm our PM-mediated SASP hypothesis for the incidence of respiratory diseases.

It has been widely reported that PM_2.5_ is more toxic than PM_10_ because PM_2.5_ can be breathed into the deeper parts of the lung due to its smaller size [61]. Moreover, PM_2.5_ can carry more toxic materials because it has a smaller diameter and larger surface area in the same amount [61]. In accordance with previous reports, PM_2.5_ induced severe cellular senescence with more dramatic changes in the ATM**–**p53**–**p21 signaling cascades. The more detrimental changes appeared to result from the ability of PM_2.5_ to produce more excessive ROS. Therefore, it was speculated that PM_2.5_ could generate more ROS than PM_10_ because it has a stronger cell-penetrating ability and carries more toxic materials due to its smaller size.

We hypothesized that antioxidants could prevent PM-IS in lung fibroblasts as A549 cells with ROS-suppressing abilities were resistant to PM-IS. Therefore, we examined several antioxidants, including NAC, VitC, VitD, and VitE, to identify those that prevent PM-IS in lung fibroblasts. NAC and VitC, but not VitD and VitE, reduced PM-induced excessive ROS production in cells. This result may be explained by the aqueous solubility of NAC and VitC. VitD and VitE are fat-soluble vitamins that function as antioxidants by inhibiting lipid membrane peroxidation [[62], [63], [64]]. In contrast, NAC and VitC are water-soluble antioxidants that alleviate oxidative stress by detoxifying ROS within the cells or bloodstream. The success of only water-soluble antioxidants indicated that PM-IS occurred because of excessive cytosolic ROS production rather than lipid membrane peroxidation [63,65]. Notably, NAC and VitC almost completely removed the ROS produced by PM_10_ treatment but partially reduced PM_2.5_-produced ROS. Accordingly, VitC blocked PM_10_-IS in lung fibroblasts but only alleviated PM_2.5_-IS. If PM_2.5_-IS of lung cells cannot be prevented by VitC alone, additional methods would be required to relieve lung pathogenesis. For example, senolytic intervention, which selectively kills senescent cells, may be an excellent therapeutic strategy [[66], [67], [68]]. This seems promising because senolytics have been shown to mitigate COVID-19-reminiscent lung disease caused by VIS [24]. We plan to test senolytics to prevent PM_2.5_-IS-mediated lung pathogenesis in a mouse model.

## 5 Conclusion

Prolonged PM treatment induced lung fibroblast cellular senescence via ATM-p53-p21 signaling activated by ROS-mediated DNA damage. Antioxidants such as Vit C obstructed PM-IS by reducing PM-induced ROS production.

## Author contribution statement

Sein Jin: Performed the experiments; Wrote the paper.

Sung-Jin Yoon, Wang Sik Lee, Jinyoung Jeong: Contributed reagents, materials, analysis tools or data.

Na-Young Jung, Young-Jun Park, Wantae Kim: Analyzed and interpreted the data.

Doo-Byoung Oh: Conceived and designed the experiments; Wrote the paper.

Jinho Seo: Conceived and designed the experiments; Analyzed and interpreted the data; Wrote the paper.

## Funding statement

Dr. Jinho Seo was supported by 10.13039/501100014188Ministry of Science and ICT, South Korea [NRF-2020R1C1C1006833 and CRC22011-300].

Young-Jun Park was supported by KRIBB Research Initiative Program, South Korea [KGM5322214].

## Data availability statement

No data was used for the research described in the article.

## Declaration of interest's statement

The authors declare no conflict of interest.

## Abbreviations

Particulate matter: PM; polycyclic aromatic hydrocarbons: PAH; chronic obstructive pulmonary disease: COPD; World Health Organization: WHO; senescence-associated secretory phenotype: SASP; virus-induced senescence: VIS; severe acute respiratory syndrome-coronavirus-2: SARS-CoV-2; severe coronavirus disease 2019: COVID-19; p21^Cip1/WAF1^: p21; p16^INK4a^: p16; cyclin-dependent kinase: CDK; A549: Lung adenocarcinoma cell; BEAS-2B: Lung epithelial cell line; vitamin C: VitC; N-acetyl-L-cysteine: NAC; HFL-1, IMR-90, and WI-38: Lung fibroblasts; senescence-associated β-galactosidase: SA-β-gal; reverse transcription-quantitative real-time polymerase chain reaction: RT-qPCR; interleukin: IL; C–C motif chemokine ligand 2: CCL2; matrix metalloproteinase: MMP; tumor necrosis factor: TNF; PM-induced senescence: PM-IS; phosphorylated pRB: p-pRB; phosphorylated-H2AX: γH2AX; DNA damage response: DDR; DNA double-strand break: DSB; Ataxia telangiectasia mutated: ATM; aryl hydrocarbon receptor: AHR; reactive oxygen species: ROS; chloromethyl derivative of H2DCFDA: CM-H2DCFDA; vitamin D: VitD; vitamin E: VitE; european reference material: ERM; phosphate-buffered saline: PBS; horseradish peroxidase: HRP; glyceraldehyde‐3‐phosphate dehydrogenase: GAPDH.
