# Supplementary material for: Antioxidants prevent particulate matter-induced senescence of lung fibroblasts
Source: Heliyon. 2023 Mar 1;9(3):e14179. doi: 10.1016/j.heliyon.2023.e14179 (PMC10006845; doi:10.1016/j.heliyon.2023.e14179)
Supplement: Supplementary Figures [file mmc1.docx]

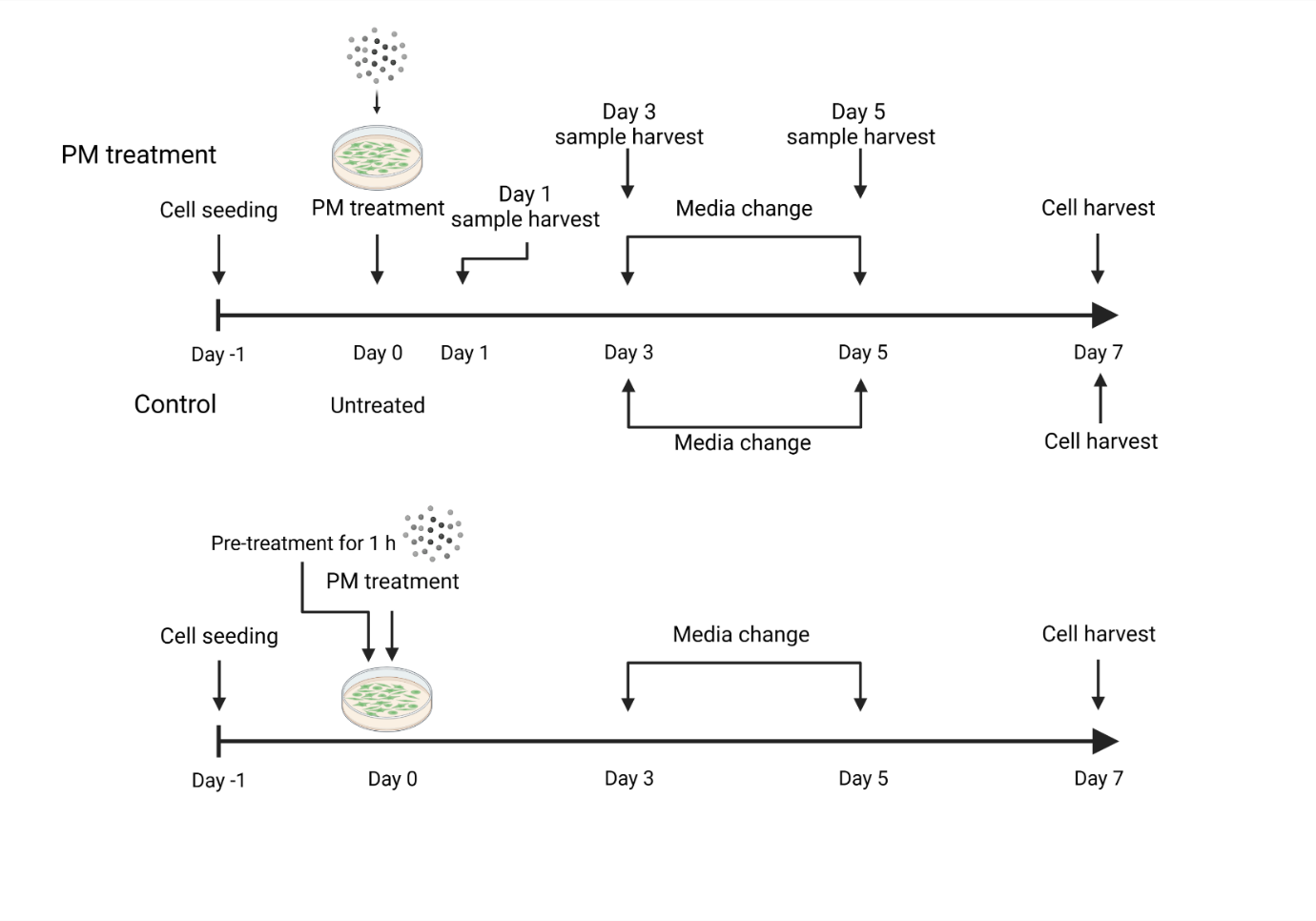


**Fig. S1. Schematic diagram of the particulate matter (PM) treatment experiments.**

**Fig. S2. N-acetyl-L-cysteine (NAC) and vitamin C (VitC), but not VitD and VitE, remove PM-induced excess reactive oxygen species (ROS) in lung fibroblasts.** HFL-1 cells were incubated with 1 mM NAC (A), 100 μM VitC (B), 100 μM VitE (C), and 100 nM VitD (D) for 1 h, followed by treatment with 10 μg/cm^2^ of PM_10_ or PM_2.5_ for 7 days. ROS was analyzed via chloromethyl derivative of H_2_DCFDA (CM-H_2_DCFDA) staining-based (10 μM) flow cytometry.

**Fig. S3. Images of western blot raw data (Fig. 3A)**

**Fig. S4. Images of western blot raw data (Fig. 4B)**

**Fig. S5. Images of western blot raw data (Fig. 6A)**

**Fig. S6. Images of western blot raw data (Fig. 7E)**
